# Critical Risk Assessment, Diagnosis, and Survival Analysis of Breast Cancer

**DOI:** 10.3390/diagnostics14100984

**Published:** 2024-05-08

**Authors:** Shamiha Binta Manir, Priya Deshpande

**Affiliations:** Department of EECE, Marquette University, Milwaukee, WI 53233, USA; priya.deshpande@marquette.edu

**Keywords:** resampling, principal component analysis, random forest, K-nearest neighbors, logistic regression

## Abstract

Breast cancer is the most prevalent type of cancer in women. Risk factor assessment can aid in directing counseling regarding risk reduction and breast cancer surveillance. This research aims to (1) investigate the relationship between various risk factors and breast cancer incidence using the BCSC (Breast Cancer Surveillance Consortium) Risk Factor Dataset and create a prediction model for assessing the risk of developing breast cancer; (2) diagnose breast cancer using the Breast Cancer Wisconsin diagnostic dataset; and (3) analyze breast cancer survivability using the SEER (Surveillance, Epidemiology, and End Results) Breast Cancer Dataset. Applying resampling techniques on the training dataset before using various machine learning techniques can affect the performance of the classifiers. The three breast cancer datasets were examined using a variety of pre-processing approaches and classification models to assess their performance in terms of accuracy, precision, F-1 scores, etc. The PCA (principal component analysis) and resampling strategies produced remarkable results. For the BCSC Dataset, the Random Forest algorithm exhibited the best performance out of the applied classifiers, with an accuracy of 87.53%. Out of the different resampling techniques applied to the training dataset for training the Random Forest classifier, the Tomek Link exhibited the best test accuracy, at 87.47%. We compared all the models used with previously used techniques. After applying the resampling techniques, the accuracy scores of the test data decreased even if the training data accuracy increased. For the Breast Cancer Wisconsin diagnostic dataset, the K-Nearest Neighbor algorithm had the best accuracy with the original dataset test set, at 94.71%, and the PCA dataset test set exhibited 95.29% accuracy for detecting breast cancer. Using the SEER Dataset, this study also explores survival analysis, employing supervised and unsupervised learning approaches to offer insights into the variables affecting breast cancer survivability. This study emphasizes the significance of individualized approaches in the management and treatment of breast cancer by incorporating phenotypic variations and recognizing the heterogeneity of the disease. Through data-driven insights and advanced machine learning, this study contributes significantly to the ongoing efforts in breast cancer research, diagnostics, and personalized medicine.

## 1. Introduction

Breast cancer is a significant public health concern among women. According to the American Cancer Society, high body weight, physical inactivity, and alcohol consumption are risk factors that contribute to 30% of breast cancer cases [1]. Even while these variables may be changed to potentially lower risk, determining a woman’s unique risk profile is crucial for directing counseling regarding risk reduction, genetic testing, and breast cancer surveillance.

In recent years, substantial advancements have been made in the risk assessment, diagnosis, and treatment of breast cancer. Early detection significantly improves survival rates, with a 99% 5-year relative survival rate for localized cases [2]. Consequently, accurate risk assessment is crucial to determining whether a woman has an average or elevated chance of developing breast cancer. Women at different risk levels can benefit from customized management techniques based on this categorization [3].

This study’s main goal is to improve knowledge about breast cancer and how it is managed by employing a thorough and multifaceted approach. Through the utilization of large-scale datasets and machine learning skills, our goal is to investigate multiple aspects of breast cancer research, including risk evaluation, improved diagnosis, and a detailed examination of survivability variables. This study also focuses on breast cancer risk prediction, identifying individuals at varying risk levels by integrating multiple risk factors into our models to facilitate early diagnosis [4].

It is worth noting that artificial intelligence (AI) systems have shown the ability to improve breast cancer diagnosis by outperforming traditional radiological methods by 11.5% [5]. Consequently, there is an urgent need for enhanced automated screening and diagnosis procedures. Using the Breast Cancer Wisconsin (diagnostic) dataset, this study demonstrates the application of diverse machine learning algorithms to the task of breast cancer diagnosis.

Furthermore, the impact of breast cancer is not uniform across all demographic groups, leading to clusters with varying incidence and mortality rates [6]. Effective screening and prognostic identification are pivotal in addressing this heterogeneity. The objective is to determine and depict relevant demographic and prognostic characteristics that affect breast cancer survivability by using machine learning techniques. Breast cancer survivability rates highlight the value of routine screening [7]. The SEER Breast Cancer Dataset enables a thorough analysis of survivability from both supervised and unsupervised perspectives.

Several studies have contributed significantly to breast cancer diagnosis, risk assessment, and survivability analysis. The literature review that follows focuses on several significant studies that provide insight into risk factors, predictive models, and diagnostic precision in breast cancer research.

Kabir et al. conducted experiments on a breast cancer dataset with imbalanced data, using various resampling techniques to adjust the training data. They used resampling techniques like random undersampling (RUS) of the majority class, random oversampling (ROS) of the minority class, synthetic minority oversampling technique (SMOTE), edited nearest neighbors (ENN), SMOTE combined with ENN, and SMOTE combined with Tomek Link. They also used Decision Trees (DTs), Random Forests (RFs), and extreme gradient boosting (XGBoost) classifiers. The results showed that using resampling techniques improved performance, particularly for the minority class. The highest accuracy achieved for DT was 90.69% with ENN, for RF it was 88.55% with SMOTE + ENN, and for XGBoost it was 91.49% with ENN. However, the overall performance was better without applying any resampling method for the minority class [8].

Using information gathered during screening, Louro et al. created a risk prediction model to estimate the short- and long-term risk of breast cancer in women undergoing mammography. They used partially conditional Cox proportional hazard regression including covariates like age, family history, past benign breast disease, and mammographic characteristics. Over a period of two to twenty years, the E/O ratio (the ratio of expected to observed cases in the target group) varied from 0.99 to 1.02. The 4-year risk estimate had the lowest AUC (area under the curve) (58.7%, 95% CI: 55.9–61.5%) and the 18-year risk estimate had the highest AUC (64.7%, 95% CI: 62.5–66.9%) [9]. 

Behravan et al. proposed a method that used the XGBoost machine learning model with an adaptive iterative search algorithm to identify the interactions between genetic and demographic risk factors that provide the best accuracy in predicting breast cancer (BC). The first module provides a list of potential BC-risk-predictive traits, while the second module captures the interacting characteristics that produce the best BC risk prediction accuracy on validation data. By combining interacting genetic features and family history features, the proposed approach achieved a mean average precision (mAP) of 77.78 on the Kuopio Breast Cancer Project (KBCP) dataset, which was better than the mAPs of 74.19 and 73.65 obtained by using only Group 1 features and interacting SNPs, respectively. When using only estrogen metabolism (Group 2) features, the mAP was 72.57, but by combining interacting genetic and Group 2 features, the mAP increased to 78.00, outperforming the former [10]. 

In 2021, Gupta et al. investigated the effects of several class balancing methods on models created from an unbalanced mammographic dataset. In 2022, Sood et al. created a breast cancer prognostic modeling method using the minimum necessary data from mammography screening [11]. 

In 2022, Sood et al. created a breast cancer prognostic modeling method using the minimum necessary data from mammography screening. The paper discussed the use of machine learning techniques for predicting breast cancer in women. The researchers experimented with seven different machine learning algorithms, including Naive Bayes, SVM, K-Nearest Neighbors, Decision Trees, Random Forests, Adaboost, and deep learning. They evaluated the performance of these algorithms based on their ability to correctly identify cancerous and noncancerous cases. The researchers noted a significant improvement in the accuracy of the models after addressing the imbalance in the data through resampling techniques. The best accuracy achieved was 92.46% on the actual datasets [12].

Lavanya et al. conducted a study on multiple breast cancer datasets to evaluate the performance of the CART (Classification and Regression Tree) Decision Tree classifier with and without feature selection. The outcomes demonstrated that a certain feature selection made using the CART had improved the classification precision of a specific dataset [13]. The study by Naji et al. utilized the Breast Cancer Wisconsin diagnostic dataset and five machine learning algorithms, namely, Support Vector Machines, Random Forests, Logistic Regression, Decision Trees, and K-Nearest Neighbors, to accurately predict and diagnose breast cancer [14]. The research by Salama et al. uses accuracy in classification and confusion matrices to compare the performance of various classifiers (Decision Trees, Multi-Layer Perception, Naive Bayes, Sequential Minimal Optimization, and Instance-Based for K-Nearest Neighbors) on three separate sets of data of breast cancer. In order to obtain the best multi-classifier strategy for each dataset, the study also introduces a combination at the classification level among these classifiers [15]. The accuracy of various deep learning algorithms for predicting breast cancer patients’ post-operative survival is compared in the research by Gupta et al. Artificial neural networks, restricted Boltzmann machines, deep autoencoders, and convolutional neural networks (CNNs) are some of the techniques that were investigated [16].

Our paper on breast cancer research is informed by a range of methodologies and perspectives, as outlined in this literature review. This comprehensive approach supports our efforts to thoroughly address the topic.

The paper adheres to a conventional structure; Section 2 and Section 3 describe the methodology and the analysis of the results obtained, respectively. Finally, Section 4 discusses future directions and concludes the research.

## 2. Methodology

### 2.1. Risk Assessment Using BCSC Risk Factor Dataset

The BCSC (Breast Cancer Surveillance Consortium) Risk Factor Dataset includes risk factor data from mammograms performed at the BCSC between January 2005 and December 2017. To build this dataset, one exam per woman per calendar year and per age was chosen.

**Figure 1:**
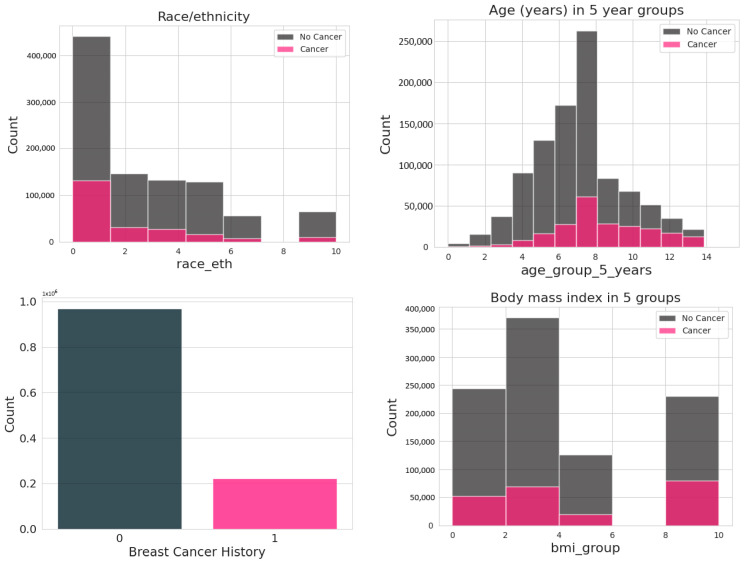
Data Visualization of the BCSC risk factor dataset.

In the data from the 6,788,436 mammograms, the 13 attributes are age, race/ethnicity, family history of breast cancer, age at menarche, age at first birth, breast density, use of hormone replacement therapy, menopausal status, body mass index, history of biopsy, and history of breast cancer. There is also an attribute named count, which shows the frequency count of that combination of features [17]. For data pre-processing, after applying several approaches like removing the year column, rows with unknown values, and data scaling the dataset, 527 k records were left (Figure 1). 

We addressed the dataset imbalance through resampling techniques—random undersampling of the majority class and oversampling of the minority class. The goal was to generate a balanced training dataset and mitigate classifier bias toward the dominant class. We then trained various classifier models using both the imbalanced and resampled training data.

The prediction performance of our models is directly affected by the data quality and relevance, which were ensured by this thorough pre-processing stage. Machine learning models that followed were able to zero in on real patterns, not data collecting artifacts or representation biases, because we scaled the dataset and corrected imbalances. Our study’s reliability is enhanced and it aligns with best practices in data science for health-related research because of this careful methodology.

The following sections provide detailed information on the selected machine learning algorithms and their performance indicators.

Step 1: First, we obtained breast cancer risk factor data for the classification. Training data contain 70% and test data contain the remaining 30% of the dataset. 

Step 2: In this step, the training data are resampled because they are of unequal distribution in the dataset. The test data are not resampled and kept the same. 

Step 3: In this step, different classifier models are trained first using the imbalanced training data. After that, we use resampled training data which had been modified by resampling methods for the classifiers. 

Step 4: Apply test data on the models to predict the risk of developing breast cancer.

**Figure 2:**
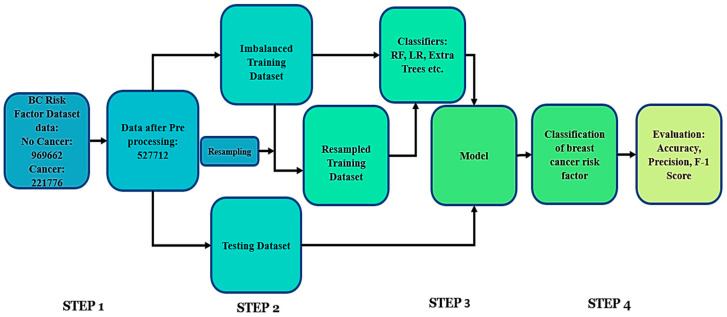
Flowchart of the proposed approach for dataset 1.

During Step 2, we used various resampling strategies to balance the BCSC Risk Factor Dataset. To balance the majority class, we randomly undersampled, and to enhance the minority class, we used the synthetic minority oversampling method (SMOTE). For Step 3’s classification problem, we employed machine learning techniques such as Decision Trees (DTs), Random Forests (RFs), and extreme gradient boosting (XGBoost), and evaluated their performance using key measures like accuracy, sensitivity, specificity, and precision (Figure 2).

### 2.2. Diagnosis Using Breast Cancer Wisconsin (Diagnostic) Dataset

**Figure 3:**
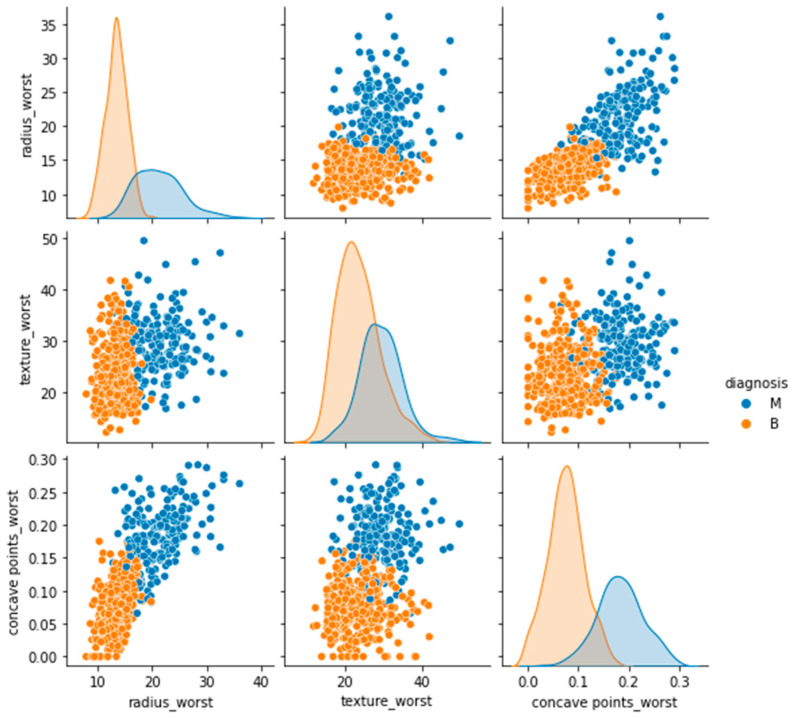
Pairwise relationship of malignant and benign tumors based on three important features from the Breast Cancer Wisconsin diagnostic dataset.

In the Breast Cancer Wisconsin (diagnostic) dataset, features were extracted to describe the characteristics of the cell nuclei from the digitized images of FNA (fine needle aspirate) biopsy. The dataset contains 569 data points, 357 benign and 212 malignant. Cell nucleus radius, texture, perimeter, area, smoothness, compactness, concavity, concave points, symmetry, and fractal dimension were the 10 features calculated in the dataset (Figure 3). Each attribute contains three data elements: the mean, the standard deviation, and the greatest or “worst” value (the mean of the three highest values), resulting in a dataset with a total of 30 features [18].

Before running the Decision Tree (DT) and K-Nearest Neighbor (KNN) algorithms, a principal component analysis was run on the dataset, and PC1 to PC7 was able to maintain more than 91% of the total variance. So, dimensionality was reduced from 30 features to 7 principal components. The efficiency of principal component analysis (PCA) in simplifying complex data without significantly reducing information loss encouraged our decision to use it. In addition to lowering computing requirements, this method lowered the risk of overfitting by zeroing in on the dataset’s most important features. More accurate and generalizable results were obtained after applying machine learning models to this optimized dataset, demonstrating the need for careful data preparation in diagnostic investigations.

Then, both the original dataset and the new principal component dataset were split into a 30% validation set and a 70% training set and 10-fold cross-validation was performed. Then, both datasets underwent classification using Decision Trees and KNN. We applied the KNN model, which uses K = 10, on the validation dataset of the original dataset and applied the KNN model, which uses K = 6, on the validation dataset of the PC dataset. The Decision Trees were pruned to minimize their size by removing the branches that could not classify cases. Best pruned and minimum error trees were found using xerror and xstd. 

These models’ performance was assessed using the criteria of accuracy, sensitivity, specificity, and precision. A receiver operating characteristic (ROC) curve was used to graphically display performance.

### 2.3. Survival Analysis Using SEER Breast Cancer Dataset

**Figure 4:**
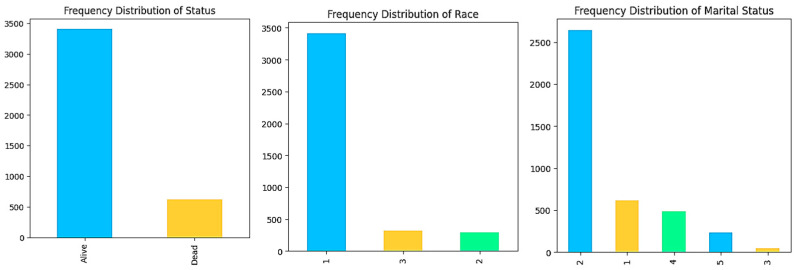
Data visualization of the SEER Breast Cancer Dataset.

The SEER Breast Cancer Dataset of breast cancer patients was made available in the NCI’s Surveillance, Epidemiology and End Results (SEER) program’s November 2017 update. SEER provides information on population-based cancer statistics. Age, race, marital status, tumor size, estrogen status, and progesterone status are some of the 15 features in this dataset. The 4024 data points in the dataset have a survivability status of 616 dead and 3408 living [19] (Figure 4).

The various categorical feature types were converted into numeric values for data pre-processing. For instance, the race categories White, Black, Other (American Indian/AK Native, Asian/Pacific Islander), and Unknown were denoted by 1, 2, 3, and 4, respectively. Similarly, positive estrogen progesterone status was represented as 1, and negative estrogen progesterone status was represented as 2. This was performed for all the feature columns. The survivability status, either alive or dead, was transformed to 1 s and 0 s. After that, dealing with missing values, scaling the data, and eliminating duplicate data and unnecessary columns from the dataset were all steps of preparing the data.

In order to prepare the dataset for advanced machine learning analysis, it was necessary to transform categorical features into numerical values and fix any missing data. The accuracy of our models in representing the complicated facts of breast cancer survival outcomes is ensured by these processes, which involve interpreting and analyzing the data. Our rigorous methodology lays the groundwork for investigating the interplay of survivability-related variables, demonstrating the promise of machine learning as a tool for improving patient care.

Unsupervised data mining techniques such as cluster analysis can be used to divide the patients into groups to determine which factors are more strongly associated with breast cancer survival [20]. In this project, different clustering methods, including agglomerative hierarchical clustering and K-means clustering, were used to find similarities between the patient features for breast cancer survivability. Data points are organized using K-means into discrete, non-overlapping groupings. So, K-means clustering was one of the machine learning algorithms used in this project. Tree structures can be created from related datasets using hierarchical clustering techniques such as AGNES. The relationships between various sub-clusters and the distances between data points are visible. For both clustering techniques in R, random sample sets from the data were taken, and then clustering was applied to sample data. As the sample sets were selected randomly, they can be considered a fairly accurate representation of the whole dataset.

Clustering, an unsupervised method, is frequently combined with other kinds of analysis. Two supervised learning techniques were used: Logistic Regression and the Decision Tree algorithm. Logistic Regression was used as the outcome of survivability status was binary: either dead or alive. For the Logistic Regression algorithm, data were divided into a test set and a training set, with 70% of the dataset going into the training set and 30% going into the test set. The Decision Trees were pruned to minimize their size by removing the branches that could not classify cases. Best pruned and minimum error trees were found using xerror and xstd. Time series cross-validation was employed to forecast the survivability of breast cancer patients. The time series was based on survival months and was created using complete dates, including days. There were 107 months of survival data.

## 3. Results

### 3.1. Results of Risk Assessment

**Figure 5:**
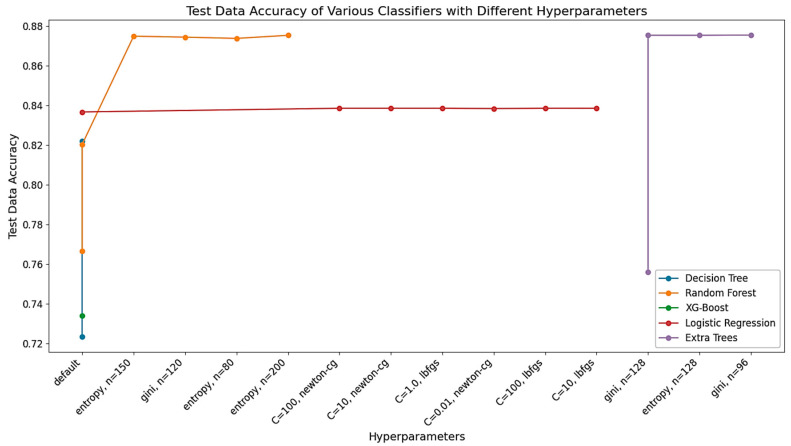
Comparison of classifier performance on the BCSC Dataset.

To evaluate the performance of the different classification techniques applied to the BCSC Dataset, such as Random Forests, Logistic Regression, Extra Trees, etc., the accuracy, precision, and F-1 scores were derived. The accuracy scores are given in Table 1 and Figure 5.

With respect to their individual hyperparameter settings, the test data accuracy of several classifiers used on the Breast Cancer Surveillance Consortium (BCSC) Dataset is shown in this figure. Different hyperparameter configurations, including g‘ini, n = 128’, e‘ntropy, n = 150’, and others, were tested for each classifier, which includes Decision Trees, Random Forests, XG-Boost, Logistic Regression, and Extra Trees. In addition to demonstrating the influence of hyperparameter modification on model performance, the graph attempts to visually evaluate how well these classifiers predict the risk of breast cancer. Finding the most accurate model configurations to improve the prediction accuracy in the assessment of breast cancer risk is dependent on this comparison.

Table 2 shows the accuracy of the Random Forest classifier after applying the resampling techniques SMOTE, RUS, ROS, and Tomek Link. The Random Forest algorithm had the best performance out of all the classifiers with an accuracy of 87.53%. Out of the different resampling techniques applied to the training dataset for using it to train the Random Forest classifier, the Tomek Link had the best test accuracy, at 87.47%.

The BCSC Dataset was used to evaluate various classification techniques, including Decision Trees, Random Forests, XG-Boost, Logistic Regression, and Extra Trees. The accuracy of each classifier was measured using both training and test data to determine how well the models learned from the training data and generalized to new data. The Decision Tree and Random Forest models had a high training data accuracy, but a notable overfitting issue was observed. The XG-Boost model had comparatively lower accuracy scores, while the Logistic Regression model performed well on both the training and test data. The Extra Trees classifier showed promising results after fine-tuning with specific hyperparameters.

As an example, while the Decision Tree classifier achieved a training data accuracy of 97.1%, its test data accuracy of 72.3% indicates that it may struggle to generalize to new data points. On the other hand, the Random Forest classifier achieved a similar training data accuracy but demonstrated a better generalization ability, with a higher test data accuracy of 76.6%. These discrepancies in accuracy can be attributed to the inherent complexity of the models and their susceptibility to overfitting or underfitting.

The Tomek Link approach improved the Random Forest classifier’s accuracy by removing noisy and ambiguous examples from the training data, resulting in better class distinction and more accurate predictions.

### 3.2. Results of Diagnosis

The overall performance of the classifiers on the Breast Cancer Wisconsin diagnostic datasets is given in Table 3. From this, we can see that the KNN classifier exhibited the best accuracy out of the three. And in the case of the dataset, the classifiers had a better accuracy, sensitivity, specificity, and precision for the validation set by being trained with the seven-principal-component dataset training set rather than the original dataset training set. 

From the figures in Table 4, we can see lift charts and ROC curves for KNN and DT. This leads us to better understand the performance of the models. For example, in the decile-wise lift chart of the Decision Tree model for the principal component dataset, the lift value of the leftmost bar is 2.5, meaning that for the top 35% of the validation cases with the highest predicted probability of belonging to the target class, the model would identify 2.5 times as many target-class cases than if the cases were randomly selected.

An evaluation method for binary classification issues is the Receiver Operator Characteristic (ROC) curve. The values for the area under the curve (AUC) are as follows: Logistic Regression AUC = 0.9344, KNN AUC = 0.9868, KNN with PCA AUC = 0.9796, DT AUC = 0.8826, DT with PCA AUC = 0.9302. Having a higher AUC means the model is more accurate at classifying zero classes as 0 and classifying one class as 1.

Using the PCA dataset reduced the dimensionality of the dataset to less than one-third (from 30 features to 7) and retained more than 91% of the information of the original dataset. Although using PCA reduces the interpretability of a dataset, it reduces the collinearity of the dataset by taking highly correlated variables and turning them into uncorrelated variables. Other types of ensemble trees (such as Random Forests) could have been used for the classification model, but as they are a combination of several Decision Trees, they need rigorous training and a longer processing time. The use of Logistic Regression can be justified because the classification problem is binary, that is, it diagnoses whether a sample tumor cell from the FNA is benign (noncancerous) or malignant (cancerous).

### 3.3. Results of Survival Analysis

The agglomerative hierarchical clustering Ward method was used on the SEER Breast Cancer Dataset, and the agglomerative coefficient was 0.981667. Clustering was performed on a sample dataset of 50.

**Figure 6:**
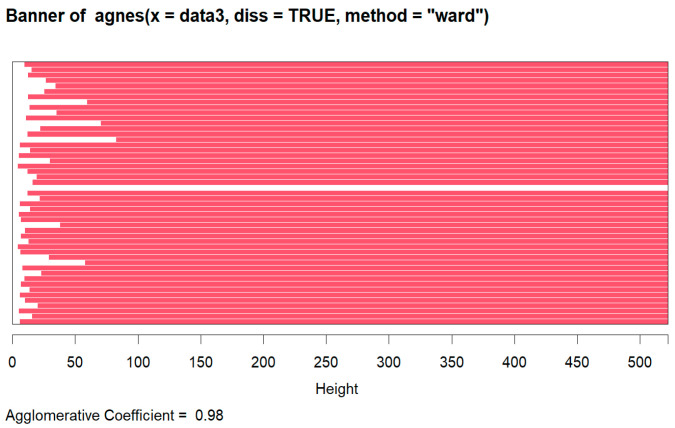
Banner plot made using AGNES.

**Figure 7:**
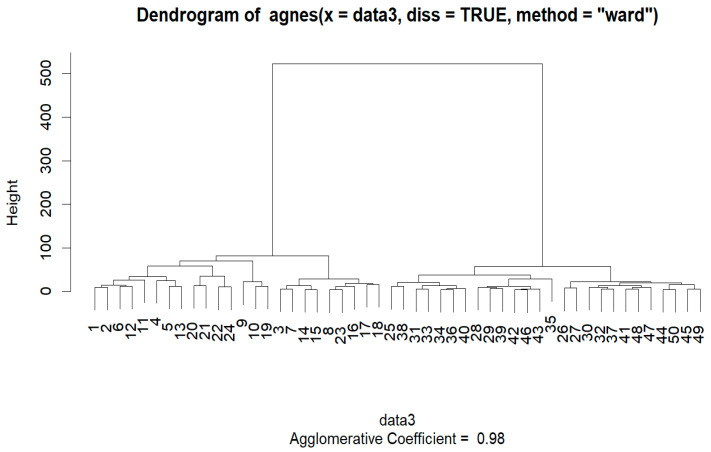
Dendrogram made using AGNES.

A graphic representation known as a “banner plot” was created during the AGNES clustering procedure. In this diagram, the observations are displayed as red bars, with spaces between them denoting possible clusters. The banner plot sheds light on cluster formation by highlighting areas of separation, similar to the idea of cutting a dendrogram to obtain the desired amount of clusters (Figure 6 and Figure 7).

**Figure 8:**
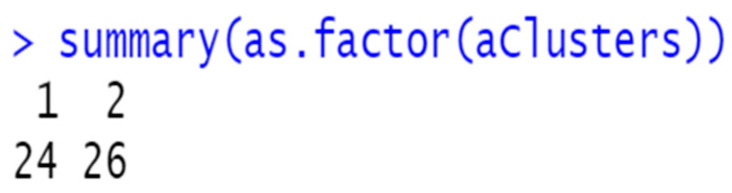
Summary of K-means cluster analysis.

**Figure 9:**
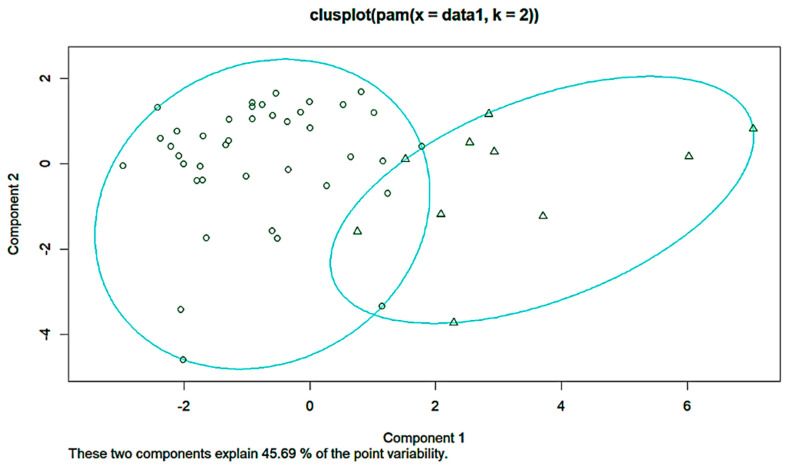
Cluster plot of K-means.

**Figure 10:**
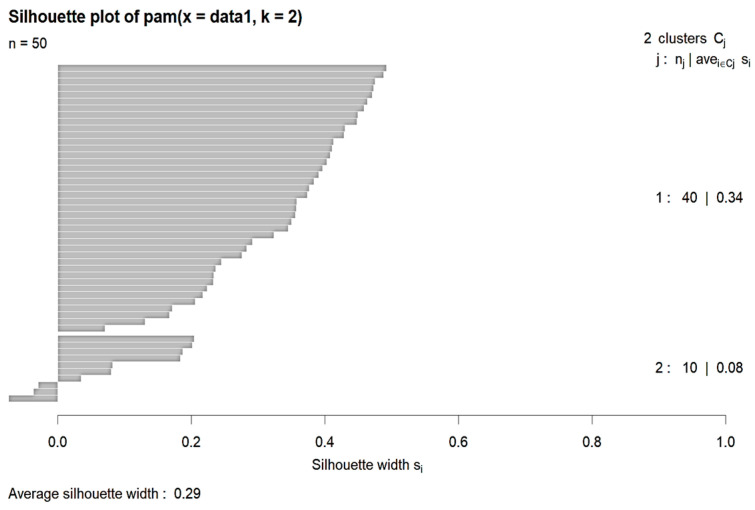
Silhouette plot of K-means.

From observing the banner plot, K was set to 2. The summary of the cluster analysis is as follows (Figure 8):

K-means clustering was performed for comparison. Here, again, K was set to 2. The following are the cluster plot and silhouette plot showing the results of the K-means clustering (Figure 9 and Figure 10).

Two types of supervised learning techniques, Logistic Regression and Decision Trees, were used for predicting the survivability of breast cancer. The whole dataset was used for this. The overall performance of the classifiers on the datasets is given in Table 5.

**Figure 11:**
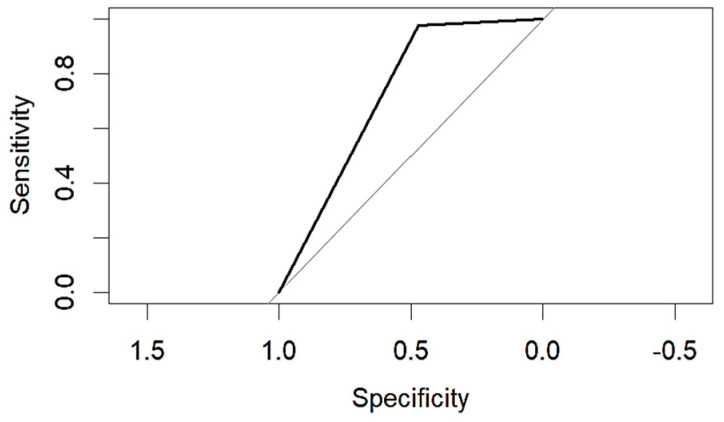
ROC of LR.

**Figure 12:**
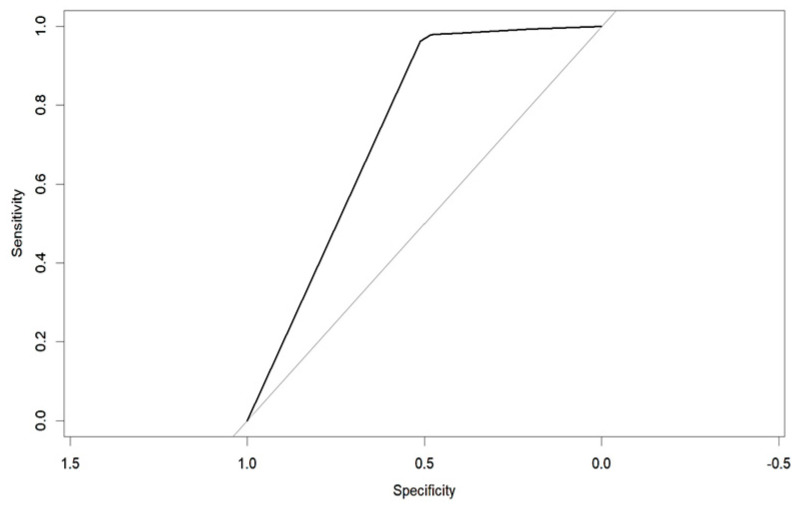
ROC of DT.

**Figure 13:**
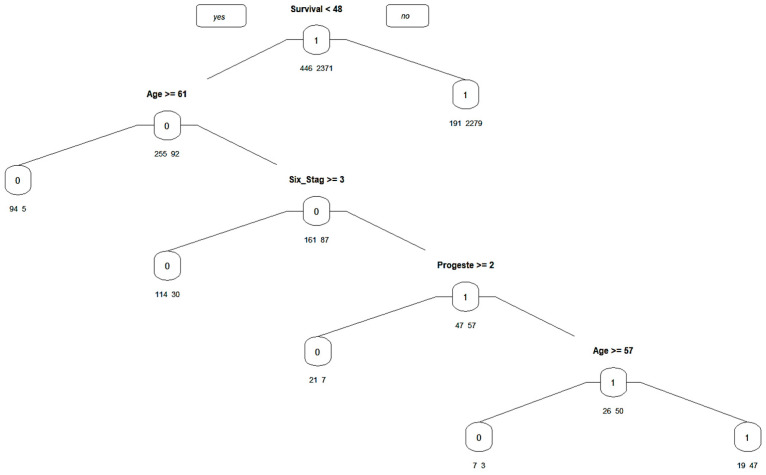
Decision tree.

The table shows that the Decision Tree classifier has a better accuracy and a larger area under the curve than Logistic Regression. The ROC curve for both LR and DT are given in the following Figure 11 and Figure 12: 

The pruned Decision Tree is given below (Figure 13):

Finally, a forecasting method, time series cross-validation, was used. The time series was survivability months. The performance measures are given in Table 6.

A data pre-processing technique such as PCA was not used as the dataset only had 15 columns. For applying the forecasting methods, it would be best to use a dataset related to breast cancer survivability over time. Although we used the SEER BC Dataset for time series cross-validation, it is not entirely appropriate for forecasting. Other ensemble tree types, like Random Forest, may have been used for the classification model. Still, because they combine multiple Decision Trees, they require extensive training and take longer to process.

## 4. Discussion

In this study, we addressed the challenge of unbalanced data in breast cancer risk factor assessment by employing various classifiers, including Decision Trees (DTs), Random Forests (RFs), Extreme Gradient Boosting (XGBoost), Support Vector Machines, and more. Although the training data accuracy was considerable, there is room for much improvement regarding the test data accuracy. Our findings, based on a thorough evaluation of the current literature, contribute to an improved understanding of machine learning applications in breast cancer risk assessment and diagnosis. Previous research by Kabir et al. [8] and Gupta et al. [11] has highlighted the impact of resampling techniques on classifier performance, but our study delves deeper into the specific effectiveness of techniques such as Tomek Link in improving Random Forest classifier outcomes for breast cancer datasets.

After applying the resampling techniques, the accuracy scores of the test data decreased even if the training data accuracy increased. This paradox highlights the nuanced challenge of balancing model generalizability and overfitting, a key area where our study contributes to the conversation beyond the findings of Louro et al. [9] and Behravan et al. [10], who emphasized predictive accuracy without a strong focus on the balancing act required for generalizable model performance.

The goal is to build better models that can predict more accurately and provide a better performance. In the future, we want to find out how different biases in the dataset toward age, race, and BMI index can affect the classification techniques and find out which of the variables has the most impact on predicting the risk of developing breast cancer.

According to the World Health Organization (WHO), 2.3 million women were diagnosed with breast cancer in 2020 globally. The use of machine learning algorithms for diagnosing breast cancer can improve the efficiency of the diagnosis and help with early detection. By addressing a gap in the use of computer-aided diagnosis systems as described by McKinney et al. [5], our comparative examination of machine learning algorithms indicates the potential for dramatically reducing the high percentage of errors and inconsistencies in radiology practice.

For this project, Logistic Regression, KNN, and Decision Tree algorithms were used with several data pre-processing techniques. The aim was for the models to classify the incidence of breast cancer from the dataset of FNA biopsy image features. This multidisciplinary approach not only improves the diagnostic process, but it also provides a road map for incorporating machine learning into clinical processes, as proposed by the scientific community.

Early precautions can be taken if demographic factors indicating high risk of breast cancer mortality are identified. It can be used to direct management for women with all breast cancer stages. Our study into the impact of demographic parameters on model accuracy and survival analysis adds to a more tailored, data-driven approach to breast cancer management, which represents a significant advancement in the field.

Defining a similarity measure between patient data is a crucial first step to stratifying patients into clinically significant subgroups and enabling individualized therapy. By utilizing advanced machine learning techniques, our research lays the groundwork for future studies to investigate individualized treatment strategies based on breast cancer’s phenotypic differences, addressing the need for innovation in the personalized medicine environment.

The results must be thoroughly scrutinized for accuracy when discussing a subject like cancer, so trying different types of prediction and classification techniques would help in the diagnostic process. This study, therefore, not only presents a comprehensive assessment of machine learning’s role in breast cancer research but also sets the stage for future innovations that can further refine predictive models, making them more adaptable and accurate.

Lastly, we highlight the novel characteristics of our machine learning approach to breast cancer research by placing our results in the context of the larger literature and emphasizing the particular contributions of our study. By laying the groundwork for future research that may fully utilize machine learning in oncology, our study advances the continuous development of more efficient, individualized cancer care solutions.

## 5. Conclusions

To summarize, this study highlights the significant capabilities of machine learning in assessing and diagnosing breast cancer risk. Additionally, it tackles important challenges such as imbalanced data and the ability of the model to be applied to different scenarios. Our comparative investigation has demonstrated that the utilization of unique resampling strategies, particularly the Tomek Link method, leads to a substantial enhancement in the performance of machine learning classifiers, such as Random Forests, when applied to breast cancer datasets.

Our research combines theoretical developments in machine learning with actual clinical applications. We argue for the integration of AI technologies in healthcare to improve diagnostic accuracy and detect diseases at an early stage. Moreover, we suggest establishing a basis for future investigations into how demographic characteristics affect the precision of models, highlighting the significance of individualized medication that takes into consideration the diverse nature of breast cancer.

This contribution seeks to expand the utilization of machine learning in the field of oncology, advocating for a patient-focused, data-oriented approach to cancer treatment. Going forward, prioritizing the improvement of these models to accurately represent the diverse patient groups they cater to is crucial, as it signifies progress in fully utilizing the possibilities of machine learning in the field of oncology.

## Figures and Tables

**Table 1:** Overall performance of the classifiers on BCSC Risk Factor Dataset.

Classifier	Hyperparameters	Training Data Accuracy	Test Data Accuracy
Decision Tree	default	0.971250087	0.723443296
Random Forest	default	0.971236011	0.766493249
XG-Boost	default	0.734065414	0.733957811
Decision Tree	default	0.860784303	0.822131966
Random Forest	default	0.860759939	0.820369645
Logistic Regression	default	0.83735429	0.836679005
Extra Trees	criterion = ‘gini’, n_estimators = 128	0.915882057	0.756212338
Extra Trees	criterion = ‘gini’, n_estimators = 128	0.93007542	0.875298457
Extra Trees	criterion = ‘entropy’, n_estimators = 128	0.93007542	0.875285824
Extra Trees	criterion = ‘gini’, n_estimators = 96	0.93007542	0.875386889
Logistic Regression	C = 100, max_iter = 500, solver = ‘newton-cg’	0.839243851	0.838536074
Logistic Regression	C = 10, max_iter = 500, solver = ‘newton-cg’	0.839243851	0.838536074
Logistic Regression	C = 1.0, max_iter = 500, solver = ‘lbfgs’	0.839243851	0.838536074
Logistic Regression	C = 0.01, max_iter = 500, solver = ‘newton-cg’	0.83928175	0.83839711
Logistic Regression	C = 100, max_iter = 500, solver = ‘lbfgs’	0.839243851	0.838536074
Logistic Regression	C = 10, max_iter = 500, solver = ‘lbfgs’	0.839251972	0.838523441
Random Forest	criterion = ‘entropy’, n_estimators = 150	0.93007542	0.874824715
Random Forest	criterion = ‘gini’, n_estimators = 120	0.93007542	0.87433834
Random Forest	criterion = ‘entropy’, n_estimators = 80	0.930067299	0.873713001
Random Forest	criterion = ‘entropy’, n_estimators = 200	0.93007542	0.875260558

**Table 2:** Performance of Random Forest classifier after various resamplings.

Pre-Processing	Training Data Accuracy	Test Data Accuracy
SMOTE	0.9364	0.8526
RUS	0.9404	0.8064
ROS	0.9362	0.8494
Tomek Link	0.9300	0.8747

**Table 3:** Overall performance of the classifiers on Breast Cancer Wisconsin diagnostic dataset.

Classification Methods	Dataset	Accuracy	Sensitivity	Specificity	Precision
LR	Original dataset	0.9415	0.9626	0.9062	0.9450
KNN	Original dataset	0.9471	0.9365	0.9533	0.9219
	Principal components dataset	0.9529	0.9365	0.9626	0.9365
DT	Original dataset	0.9294	0.8730	0.9626	0.9322
	Principal components dataset	0.9471	0.9048	0.9720	0.9500

**Table 4:** Cumulative lift charts, decile-wise lift charts, and ROC curve for KNN and DT using the Wisconsin Breast Cancer diagnostic dataset.

Classifier	Figure	Original Dataset	PC1–PC7 Dataset
KNN	Cumulative lift chart	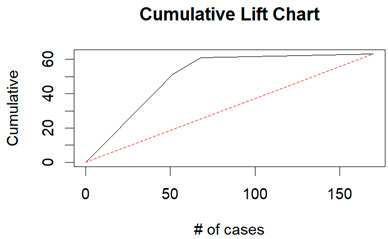	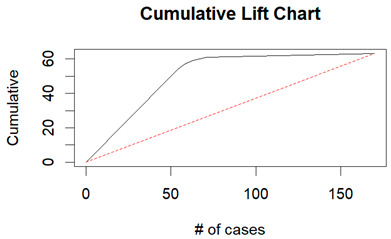
	Decile-wise lift chart	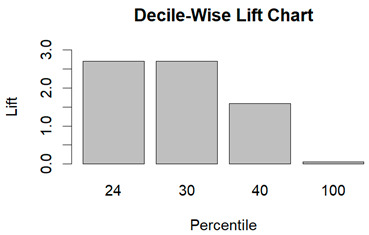	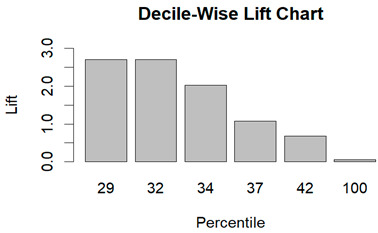
	ROC curve	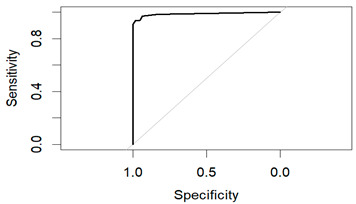	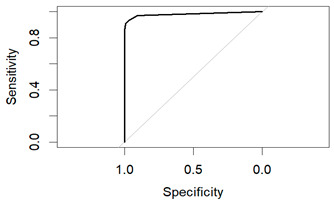
DT	Cumulative lift chart	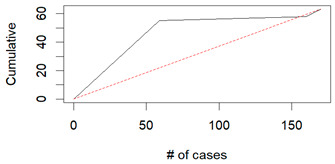	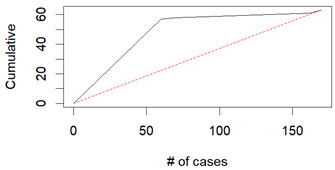
	Decile-wise lift chart	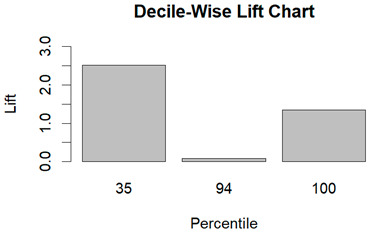	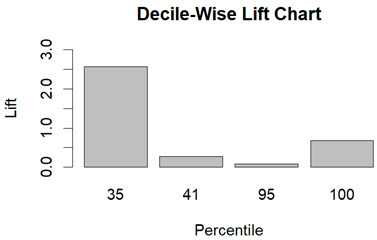
DT	ROC curve	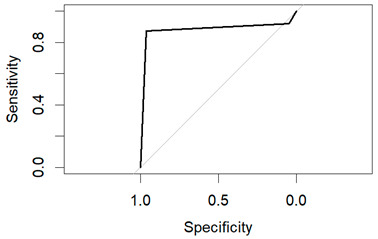	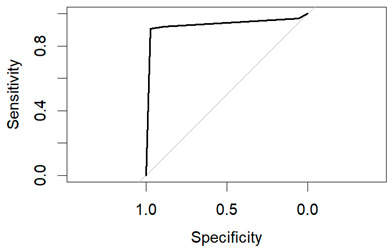

**Table 5:** Overall performance of the classifiers on SEER Breast Cancer Dataset.

Classification Method	Accuracy	Sensitivity	Specificity	Precision	AUC
LR	0.8998	0.4703	0.9775	0.7909	0.7239
DT	0.9080	0.9797	0.4706	0.9186	0.7412

**Table 6:** Performance measures of time series cross-validation.

Time Series Cross-Validation	ME	RMSE	MAE	MASE
Training set	2.141162 × 10^−17^	0.4152110	0.3448004	1.120601
Test set	2.002756 × 10^−1^	0.5176724	0.3775180	1.226933

## Data Availability

Data used in this research are publicly available. Please use the following links to access them: 1. https://www.bcsc-research.org/data/rf (accessed on 1 June 2023). 2. https://archive.ics.uci.edu/ml/datasets/breast+cancer+wisconsin+(diagnostic) (accessed on 1 June 2023). 3. https://ieee-dataport.org/open-access/seer-breast-cancer-data (accessed on 1 June 2023).
